# The neural correlates of the decoy effect in decisions

**DOI:** 10.3389/fnbeh.2014.00271

**Published:** 2014-08-07

**Authors:** Jianping Hu, Rongjun Yu

**Affiliations:** ^1^School of Psychology and Center for Studies of Psychological Application, South China Normal UniversityGuangzhou, Guangdong, China; ^2^School of Economics and Management and Scientific Laboratory of Economic Behaviors, South China Normal UniversityGuangzhou, Guangdong, China

**Keywords:** fMRI, decoy effect, decision making, salience processing, cognitive conflict

## Abstract

Human choices are remarkably susceptible to the context in which options are presented. The introduction of an inferior option (a decoy) into the choice set can make one of the original options (the target) more attractive than and the other original option (the competitor). This so called “decoy effect” represents a striking violation of the “context-invariant” axiom, yet its underlying neural mechanisms are not well understood. Here, we used a novel gambling task in conjunction with functional magnetic resonance imaging (fMRI) to elucidate its neural basis. At both the stimulus and decision phases, choice sets with decoys activated the occipital gyrus and deactivated the inferior parietal gyrus. At the decision phase, choosing the targets vs. the competitors elicited stronger anterior insula activation, suggesting that perceptual salience drives heuristic decision making in the decoy effect. Moreover, across participants, activity in anterior cingulate cortex (ACC) predicted a reduced susceptibility to the decoy effect, indicating that resisting the tendency to make heuristic decisions is taxing. Our findings highlight the power of the decoy effect in laboratory settings and document the neural mechanisms underlying the decoy effect.

## Introduction

**Figure 1:**
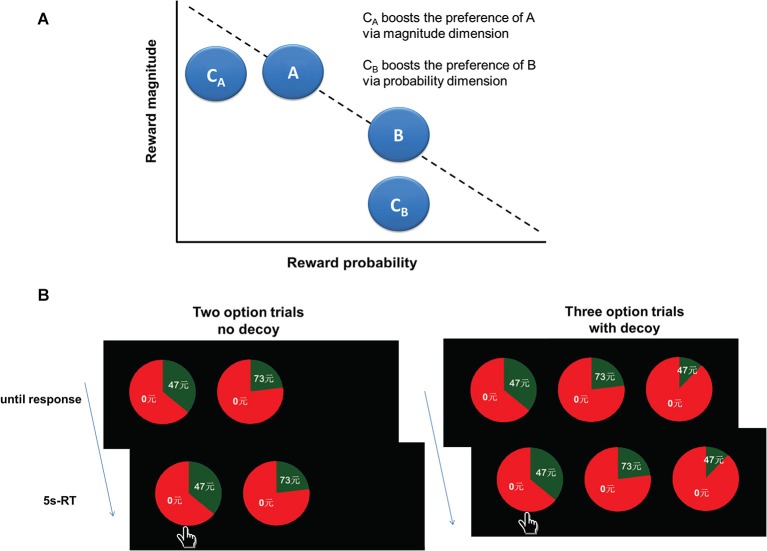
**Experimental design. (A)** A graphical illustration of the decoy effect paradigm. **(B)** Experimental task design. At the beginning of each trial, an asterisk appeared on the screen for 2 s to engage attention and eye fixation. Then participants were shown either two (two-option condition) or three gamble options (three-option condition). The gamble option was shown as a pie chart depicting the probability of winning a certain amount of money. The amounts in the gamble options varied from ￥2 to ￥92, and the probabilities varied from 1% to 86%. In the two-option condition, the expected values (EVs) of these two gamble options were equivalent. In the three-option condition, the decoy gamble options were added. The decoy gamble option had either the same amount of money or the same probability but with a smaller probability or amount of money than one of the two original gamble options, making this original gamble option the target, and the other original gamble option the competitor. The only difference between the three-option and the two-option conditions was whether the decoy option was presented or not. The two gamble options in the two-option condition were randomly presented in two of three positions. The gamble options were presented for 5 s, during which participants were instructed to make a choice by pressing 1, 2 or 3 on the keypad. Participants were also told that during the task they would not receive feedback concerning the outcomes of their decisions.

A central tenet of rational decision-making is logical consistent preference, independent of irrelevant options. However, the proposition that human decisions are “context-invariant” is challenged by a wealth of empirical data. One typical phenomenon of context-dependent preference is the decoy effect (Huber et al., 1982). As shown in Figure 1A, A is better on a given attribute (e.g., reward magnitude) but worse on another attribute (e.g., reward probability) than B. Thus, A and B are competitive to each other. A third option, such as C_A_ (termed the decoy) is added, which is similar yet inferior to A, but dissimilar to B. According to the “context-invariant” hypothesis, the inferior decoy C would not influence people’s choices between A and B. However, in reality, the decoy shifts choices toward A which is similar to the decoy and also better than the decoy. In this situation, A becomes the “target” and B the “competitor”. This phenomenon is called the decoy effect or asymmetric dominance effect.

The decoy effect has been replicated in a wide variety of choice situations involving not only commercial products (Josiam and Hobson, 1995), but also jobs (Slaughter et al., 2011) and political candidates (Pan et al., 1995). However, the neural mechanisms underlying this effect remain unclear. Hedgcock and Rao (2009) used functional magnetic resonance imaging (fMRI) to study the neural basis of the decoy effect. Greater activation in the amygdala is associated with choice sets with no decoy, as compared with choice sets enriched with decoys. They proposed trade-off aversion as an explanation of the decoy effect. Another preliminary study using magnetoencephalography (MEG) with the same paradigm revealed that right frontal areas of the brain showed neural activity differences within 750 ms when participants considered choice sets with a decoy vs. choices sets without a decoy (Hedgcock et al., 2010). The same areas of the brain had activity differences within 1000 ms when choices of the target or non-target were compared. However, the exact brain regions underlying the decoy effect are still unknown from this study due to the relatively poor spatial resolution of MEG.

Decision field theory (Roe et al., 2001) provides a detailed explanation for why target options are preferentially chosen in the decoy effect. According to decision field theory, comparing the dominated decoy (e.g., C_A_) with the other two original options (A and B) produces a negative preference state for the dominated decoy, which feeds through a negative inhibitory link to the closely positioned dominant option. Thus, the decoy makes the dominant option appear more attractive. The competitor does not experience any bolstering effect because it is too dissimilar to the dominated decoy. The pair of the target and the decoy is much more salient than the pair of the competitor and the decoy because of the similarity and difference between the target and the decoy. That is, the decoy effect occurs when the salient similarity between the target and the decoy can be detected. Similarly, Bordalo et al. (2012) proposed a salience theory to explain the decoy effect. The salience of the attribute determines the attention the decision maker pays to these attributes as well as their weight in his decision (Bordalo et al., 2012). Using eye-tracking technology and the paradigm used in the study of Hedgcock and Rao (2009) and Chen (2013) found that participants raised the rate of gazing at the decoy boosted attribute when in the choice sets with decoys compared with in the choice sets with no decoy, supporting the saliency account. Thus, salience processing might play a key role in the decoy effect. Several brain regions have been implicated in salience processing, including the anterior insula and amygdala. The anterior insula is sensitive to salient stimuli (Dehaene et al., 2001; Kuo et al., 2009) and has been considered as an important brain region in a “salience network” (Menon and Uddin, 2010). The amygdala also plays an important role in saliency processing (Santos et al., 2011).

The similarity between the target and the decoy also makes people easy to identify the dominating relationship between the two. This is the dominance heuristic account of the decoy effect (Simonson, 1989; Wedell, 1991). When participants run counter to the decoy effect, they are more likely to rely on the analytic processing instead of the heuristic processing. The heuristic process is automatic and effortless. Extra cognitive control is required to inhibit this automatic process. This may also produce a conflict between heuristic and analytic processing (De Neys, 2012; Loureiro, 2013). The anterior cingulate cortex (ACC) has been implicated in both cognitive control and conflict detection (Botvinick et al., 2004; De Martino et al., 2006; Carter and van Veen, 2007; Guo et al., 2013; Xu et al., 2013). For example, when participants ran counter to the framing effect, there was enhanced activity in the ACC (De Martino et al., 2006; Xu et al., 2013).

In the present study, we investigate neural mechanism of the decoy effect using fMRI combined with a novel gambling task (see Section Materials and Methods). We predicted that regions implicated in salience detection, heuristic decision making, cognitive control, and conflict processing may be engaged in the decoy effect.

## Materials and methods

### Participants

Sixteen healthy, right-handed volunteers (mean age and SD 24.80 ± 1.32, nine females) participated in fMRI scanning. All par­ti­ci­pants had a university degree or were in the process of obtaining one. The study was conducted with the approval of the Academic Committee of the School of South China Normal University. All participants gave written, informed consent and were informed of their right to discontinue participation at any time.

### Experimental paradigm

Before the experiment, the participants were familiarized with the decision-making task, and given practice trials. At the beginning of each trial, an asterisk was presented on the screen for 2 s to engage attention and eye fixation. Then participants were shown either two (two-option condition) or three gamble options (three-option condition). The gamble option was shown as a pie chart depicting the probability of winning a certain amount of money. The amounts of the gamble options varied from ¥2 to ¥91 and the probabilities varied from 1% to 86%. In the two-option condition, the expected values (EVs) of these two gamble options were equivalent. In the three-option condition, the decoy gamble options were added. The decoy gamble option had either the same amount of money or the same probability but with a smaller probability or amount of money than one of the two original gamble options, making this original gamble option the target, and the other original gamble option the competitor. The only difference between the three-option and the two-option conditions was whether the decoy option was presented or not. The two gamble options in the two-option condition were randomly presented in two of three positions. We also included “catch” trials to ensure that participants remained actively engaged in the decision-making task throughout the course of the experiments. In both the two-option and three-option catch trials, the expected utility of one gamble option was markedly higher than other gamble option(s). The gamble options were presented for 5 s, during which participants were instructed to make a choice by pressing 1, 2 or 3 on the keypad. Participants were also told that during the task they would not receive feedback concerning the outcomes of their decisions (see Figure 1B).

The scanning phase of the experiment lasted 24 min, which was composed of 200 trials (88 two options condition, 88 three options condition and 24 catch trials) ordered randomly. By allowing randomization across conditions, the influence of the brain responses in the previous trial on a particular type of trial was balanced. It can also minimize anticipation and habituation effects.

Participants were told that their performance in the task determined how much they would be awarded at the end of the experiment. One trial was randomly chosen and implemented. All participants received a base payment of 100 yuan (about 15 US dollars) plus any extra reward in the experiment.

### fMRI data acquisition and preprocessing

MRI scanning was conducted on a 3.0-T Siemens Allegra scanner. Whole-brain data were acquired with echo planar T2*-weighted imaging, sensitive to blood oxygenation level-dependent signal contrast (32 oblique axial slice, 3 mm thickness, 3 mm in-plane resolution; repetition time, 2000 ms, echo time, 30 ms). The imaging data were acquired at a 30° angle from the anterior commissure–posterior commissure (AC-PC) line to maximize orbital sensitivity (Deichmann et al., 2003). T1-weighted structural images were acquired at a resolution of 1 × 1 × 1 mm.

Functional image preprocessing was carried out using SPM8.^1^ To allow for equilibration effects, the first five volumes were discarded. The EPI images were sync interpolated in time for correction of slice-timing differences and realigned to the first scan by rigid-body transformations to correct for head movements. Utilizing linear and nonlinear transformations and smoothing with a Gaussian kernel of full-width-half-maximum 8 mm, EPI and structural images were co-registered and normalized to the T1 standard template in Montreal Neurological Institute (MNI) space (MNI—International Consortium for Brain Mapping). Global changes were removed by high-pass temporal filtering with a cutoff of 128 s to remove low-frequency drifts in signal.

### fMRI data analysis

Statistical analyses were performed using the general linear model (GLM). Three main types of events were distinguished: decisions in the two-option condition (two-option), targets in the three-option condition (targets), and competitors in the three-option condition (competitors). For each individual participant, two models were conducted for these events with the onsets of gamble options presentation (at the stimulus phase) and the onsets of making decisions (at the decision phase), respectively. Events were convolved with a canonical hemodynamic response function (HRF). Six head-motion parameters defined by the realignment were added to the model as regressors of no interest. Multiple linear regression was then run to generate parameter estimates for each regressor at every voxel.

In the first-level analysis, we conducted two contrasts: three-option (targets + competitors) vs. two-option and the reverse contrast at the stimulus phase; at the decision phase, we compared decisions in the following experimental conditions: two-option vs. targets, targets vs. two-option, two-option vs. competitors, competitors vs. two-option, targets chosen vs. competitors chosen, and competitors chosen vs. targets chosen. The contrast (difference in β) images of the first-level analysis were entered into one-sample *t*-test for the second-level group analysis conducted with a random effects statistical model (Penny and Holmes, 2004). The images of targets vs. competitors contrast at the decision phase were correlated with the decoy effect in behavior in a simple regression across participants.

Small volume correction (SVC) was used on a priori regions of interest including the following: the anterior insula, amygdala and the ACC, defined by the corresponding automated anatomical labeling mask (Tzourio-Mazoyer et al., 2002). Activations in other areas are reported if they survive *p* < 0.001 uncorrected, cluster size *k* > 10.

## Results

### Behavioral results

The percentage of choosing the gamble options which had markedly higher expected outcomes was 88.5% ± 10.6% (mean ± SD) in “catch” trials. Participants were highly accurate in making correct choices in these “catch” trials, providing evidence of continued engagement with the task throughout the experiment.

**Figure 2:**
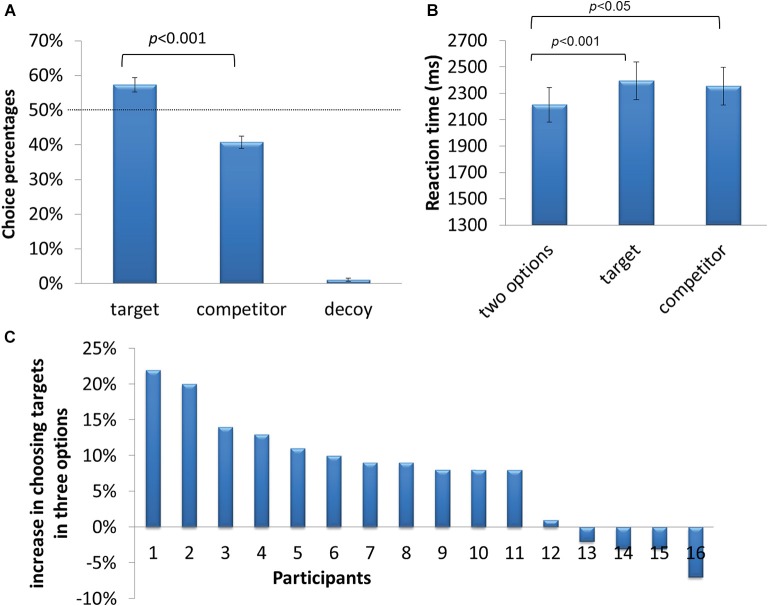
**Behavioral results. (A)** Percentage of trials in which participants chose the target, competitor and decoy. **(B)** Reaction time of choosing the target, competitor and decoy. **(C)** Individual differences in susceptibility to the decoy effect, i.e., the percentage difference between choosing the target as compared to 50%.

In the two-option condition, there was no difference between the percentages of choosing the left or right pie-chart in the two-option condition (47.5% vs. 48.9%, *t*_15_ = −0.748, *p* = 0.466). When decoys were added, participants revealed a preference for target options over the competitor options (57.3% > 40.8%, *t*_15_ = 4.316, *p* < 0.001, Figure 2A). Decoys were rarely chosen (about 1%). The behavioral results indicated that participants’ decision making processes were significantly affected by decoys. Reaction times for decision were also affected by decoys (two-option condition, 2212 ms; three-option condition, 2363 ms, *t*_15_ = −5.743, *p* < 0.001, Figure 2B). The decoy effect, defined as the frequency of choosing the target in the three-option condition minus the 50% chance level, was calculated for each participant. It varied across participants, ranging from −0.07 to 0.22 (Figure 2C).

In target-chosen and competitor-chosen trials, there was no significant difference in EVs between chosen targets and chosen competitors (10.54 vs. 11.08, *t*_15_ = 2.249, *p* = 0.154), indicating that preference for the decoy boosted targets cannot be simply explained by the differences in EVs between two experimental conditions.

## Brain activation at the stimulus phase

**Figure 3:**
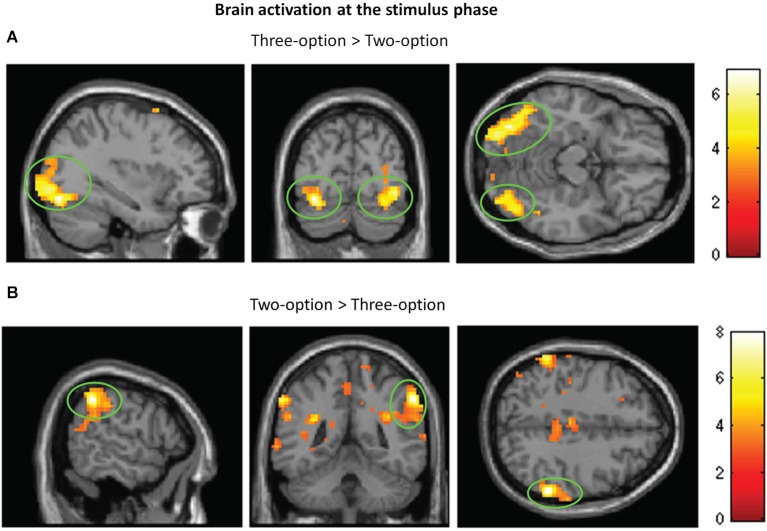
**Brain activation at the stimulus phase**. Regions with significant activation for three-option minus two-option **(A)** and for two-option minus three-option **(B)**.

At the stimulus phase, greater activity in bilateral middle occipital gyri was found in three-option condition (targets + competitors) than in two-option condition (Figure 3A; Table 1). In the reverse contrast, we observed greater activation in the inferior parietal lobule (Figure 3B; Table 1). No significant brain activation was found between targets and competitors.

**Table 1:** **Brain activation at the stimulus phase**.

**Brain Regions**	***Z*-scores**	**MNI Coordinates**		
		***X***	***Y***	***Z***
Three-option > Two-option				
L Middle Occipital Gyrus	4.56	−33	−78	−15
R Middle Occipital Gyrus	4.45	−21	−90	0
Two-option > Three-option				
R Inferior Parietal Lobule	4.90	60	−45	42
Target > Competitor				
N/A	N/A	N/A
Competitor > Target				
N/A	N/A	N/A		

## Brain activation at the decision phase

**Figure 4:**
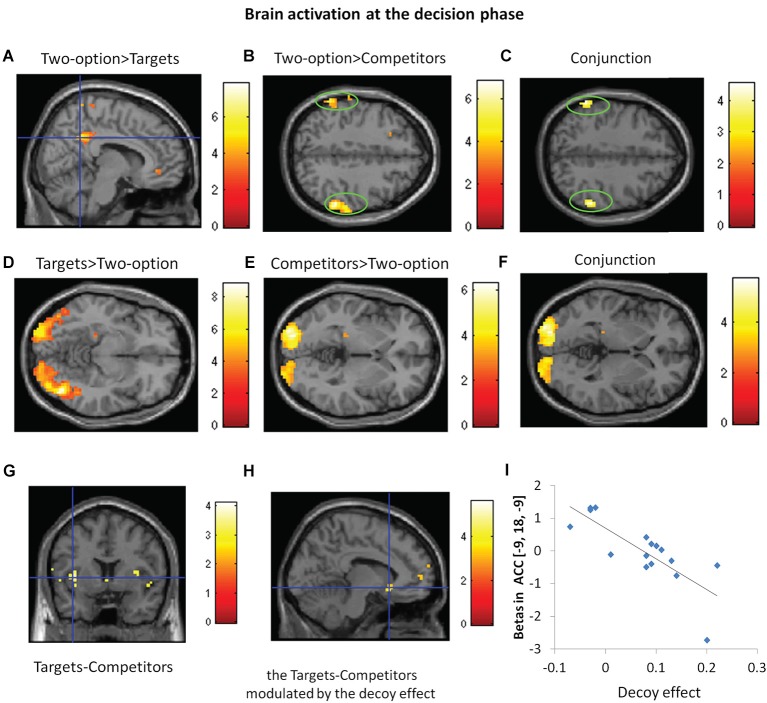
**Brain activation at the decision phase**. Regions with significant activation for responses in two-option condition (two-option) vs. targets **(A)**, two-option vs. competitors **(B)**, conjunction analysis between the contrasts of two-option vs. targets and two-option vs. competitors **(C)**, targets vs. two-option **(D)** and competitors vs. two-option **(E)**, conjunction analysis between the contrasts of targets vs. two-option and competitors vs. two-option **(F)**. **(G)** Regions with significant activation for targets vs. competitors. **(H)** Regions showing correlation between the decoy effect and ACC activation in the contrast of targets vs. competitors. **(I)** The correlation between decoy effect and parameter estimates of ACC activation for target-competitor contrast at a peak voxel (*x* = −9, *y* = 18, *z* = −9).

At the decision phase, we compared decisions in two-option trials with targets chosen condition and competitors chosen condition in the three-option trials, respectively (see Table 2). Compared with targets, two-option showed greater activation in the precuneus (Figure 4A); compared with competitors, two-option showed greater activation in the inferior parietal gyrus (Figure 4B). Conjunction analysis confirmed that two-option activated the bilateral inferior parietal gyri, compared with targets and competitors (Figure 4C).

**Table 2:** **Brain activation at the decision phase**.

**Brain Regions**	***Z*-scores**	**MNI Coordinates**		
		***X***	***Y***	***Z***
Two-option > Targets				
R Precuneus	4.13	9	−51	33
Targets > Two-option				
R Middle Occipital Gyrus	5.16	45	−72	−9
L Middle Occipital Gyrus	4.39	−24	−90	−15
Two-option > Competitors				
R Inferior Parietal Lobule	4.26	60	−42	27
Competitors > Two-option				
L Cuneus/Middle Occipital Gyrus	4.35	−21	−99	−6
Targets > Competitors				
L Anterior Insula	3.2	−39	6	3
	3.13	−36	3	12
Competitors > Targets				
N/A	N/A	N/A		

Both targets and competitors activated the middle occipital gyrus, compared with two-option (Figures 4D,E). Conjunction analysis confirmed that, targets and competitors activated the bilateral middle occipital gyri when compared with two-option (Figure 4F).

Further, we compared the brain responses in the contrasts of targets vs. competitors and the reverse contrast. Compared with choosing the competitors, choosing the targets engaged greater activity in the left anterior insula (*x* = −39, *y* = 6, *z* = 3, *Z*-score = 3.20; *x* = −36, *y* = 3, *z* = 12, *Z*-score = 3.13, *P_FWE_* < 0.05 SVC) (Figure 4G). No significant brain activation was found in the reverse contrast.

In light of the substantial inter-subject variability in behavioral susceptibility to the decoy effect, we next identified subject-specific differences in neural activity associated with their decision bias. Regression analysis revealed negative linear correlation (*r* = −0.801, *p* < 0.01) between decoy effect and the degree of activation in the left ACC (*x* = −9, *y* = 18, *z* = −9, *Z*-score = 3.63, *P*_FWE_ < 0.05 SVC) in the contrast of targets vs. competitors (Figure 4H). Individuals with a stronger decoy effect showed less activation in the ACC (Figure 4I).

## Discussion

Our fMRI data provide a neurobiological account of the decoy effect. Compared with choosing the competitors, choosing the targets engaged greater activity in the left anterior insula. Activity in the anterior insula likely reflected greater salience detection associated with choosing targets as compared with choosing the competitors. This finding is consistent with the majority of literature linking this brain region to salience processing in imaging studies (Sterzer and Kleinschmidt, 2010). For example, visible stimuli are usually more salient compared to invisible ones and are thus found to be associated with anterior insula activation (Dehaene et al., 2001). While watching an image was slowly being revealed on a screen, a sudden burst of activity in the anterior insula was found at the moment of recognition (Ploran et al., 2007). Recently, activation in the anterior insula has been associated with intuitive decision making possibly based on perceptually salient information. Intuition is the ability to understand or know something without conscious reasoning, similar to heuristics. For example, contrasting dominance-solvable games with pure coordination games, an fMRI study found that the insula is associated with extracting salient feature and facilitating the intuitive judgments in order to make an optimal choice (Kuo et al., 2009). In addition, activation within anterior insula was found in a direct contrast between intuitive and non-intuitive judgments (Volz and von Cramon, 2006).

Previous studies have also suggested an important role of the amygdala in salience processing. The amygdala is known to react to both positive and negative stimuli, with a preference for faces depicting emotional expressions (Sergerie et al., 2008; Santos et al., 2011). The lack of amygdala activation in the current study presumably could reflect the absence of emotional salience in the decoy effect. Instead, the anterior insula activation may reflect the effect of perceptual salience. This finding suggests the decoy effect may operate on perceptual process, which is consistent with a recent study of Trueblood et al. (2013). Trueblood and her colleagues found that the decoy effect can be generalized to simple perceptual task using simple perceptual stimuli.

Not everyone responds to decoy manipulations to the same degree. Our experimental design allowed us to examine for the neural basis of individual differences in susceptibility to the decoy effect. When participants’ choice ran counter to the decoy effect, there was enhanced activity in the left ACC. The ACC is believed to mediate conflict detection and to exert cognitive control during thinking (Botvinick et al., 2004; De Martino et al., 2006; De Neys et al., 2008; De Neys, 2012). For example, De Neys et al. (2008) found that, the ACC was much more activated when people solved the conflict base-rate problems than they solve the no-conflict control versions. Our results are also consistent with previous work by De Martino et al. (2006), who found enhanced activity in the ACC when participants ran counter to the framing effect. The conflict in our case is choosing competitors instead of targets, that is, abandoning the heuristic response and relying on a more analytic strategy. The enhanced ACC activation when participants were less likely to be affected by decoys may reflect the conflict detection between heuristic and analytic processes and further exerting cognitive control over the heuristic responses.

The dual-process model, proposed by Evans (2003) divides the process of decision making into contributions from two systems. System 1 is automatic, intuitive, and influenced by heuristics, whereas System 2 is deliberative, and subjective to limits of working memory. Decision making in two-option condition is more taxing than that in the three-option condition. Confronted with three-option condition, individuals compare the targets and the competitors with decoys, respectively. The anterior insula may detect the perceptually salient similarity between the decoy and the target. Such salience signal may then guide the dominance heuristic decision-making in the decoy effect. When participants rely more on the analytic strategy, they may experience the conflict between System 1 and System 2 and need extra cognitive control to inhibit the function of System 1. The more likely they run counter to the decoy effect, the more conflict they experience, possibly indicted in the enhanced activation in the ACC.

A few caveats about the present study should be mentioned. First, it is worth noting that the reaction times in situations when a decoy was available were significantly longer than RTs for decisions without a decoy. The behavioral results seem to be in odds with the idea that the decoy effect is associated with dominance heuristic. But these results also imply that when decoy effect occurs, people are taking into account comparative characteristics of alternatives. This process is driven primarily by an attempt to achieve better resolution and identify the best choice, not merely by the tendency to simplify the task (Simonson and Tversky, 1992; Roe et al., 2001). The three-option condition has more choices than the two-option condition, which might make the information processing take longer. Thus, heuristic decision making is not always faster than analytic decision making. Second, there is difficult to determine exactly when participants made their decision. For example, it could be 0.5 or 1 s before they pressed the button. We have tried using 1 s before the button press time as the onset of events and the results look similar. Since the temporal imprecision of the BLOD response is poor, it does not matter much whether the “real” decision making time is locked to decision execution or 1 s before. Previous studies have also used this “time locked to the response execution” method to successfully capture brain responses during the decision making stage (Yu et al., 2010; Fukunaga et al., 2012, 2013). Further studies may use hybrid ERP-fMRI to integrate both temporal-spatial information and further our understanding the dynamics of brain activity in decoy effect.

## Author contribution statement

Rongjun Yu conceived of the study. Jianping Hu and Rongjun Yu analyzed the data. Jianping Hu and Rongjun Yu wrote the paper.

## Conflict of interest statement

The authors declare that the research was conducted in the absence of any commercial or financial relationships that could be construed as a potential conflict of interest.

## Footnotes

^1^www.fil.ion.ucl.ac.uk/spm/
